# Methylation Profile of X-Chromosome–Related Genes in Male Breast Cancer

**DOI:** 10.3389/fonc.2020.00784

**Published:** 2020-06-17

**Authors:** Maria P. Foschini, Luca Morandi, Alejandro M. Sanchez, Angela Santoro, Antonino Mulè, Gian Franco Zannoni, Zsuzsanna Varga, Linda Moskovszky, Maria C. Cucchi, Cathy B. Moelans, Gianluca Giove, Paul J. van Diest, Riccardo Masetti

**Affiliations:** ^1^Anatomic Pathology Section “M. Malpighi”, Department of Biomedical and Neuromotor Sciences, University of Bologna, Bologna, Italy; ^2^Functional MR Unit, Department of Biomedical and Neuromotor Sciences, IRCCS Istituto delle Scienze Neurologiche di Bologna, University of Bologna, Bologna, Italy; ^3^Dipartimento Scienze della Salute della donna e del Bambino e di Sanità Pubblica, Multidisciplinary Breast Center, Fondazione Policlinico Universitario A. Gemelli IRCCS, Rome, Italy; ^4^Pathology Unit, Dipartimento Scienze della Salute della donna e del Bambino e di Sanità Pubblica, Fondazione Policlinico Universitario A. Gemelli IRCCS, Rome, Italy; ^5^Institute of Pathology and Molecular Pathology, University Hospital Zurich, Zurich, Switzerland; ^6^Unit of Breast Surgery, Department of Oncology, Bellaria Hospital, AUSL Bologna, Bologna, Italy; ^7^Department of Pathology, University Medical Center Utrecht, Utrecht University, Utrecht, Netherlands

**Keywords:** male breast cancer, androgen receptor, *MAGE* family, DNA methylation, X-chromosome, *FLNA*, *UXT*, *HDAC6*

## Abstract

**Background:** Androgen receptor (AR) has been described to play a prominent role in male breast cancer (MBC). It maps on chromosome X, and recent reports indicate that X-chromosome polysomy is frequent in MBC. Since the response to anti-androgen therapy may depend on AR polysomy and on its overexpression similarly to prostate cancer, the aim of the present study was to investigate the DNA methylation level of *AR* and its coregulators, especially those mapped on the X-chromosome, that may influence the activity of AR in MBC.

**Methods:** The DNA methylation level of *AR, MAGEA2, MAGEA11, MAGEC1, MAGEC2, FLNA, HDAC6*, and *UXT*, mapped on the X-chromosome, was evaluated by quantitative bisulfite-NGS. Bioinformatic analysis was performed in a Galaxy Project environment using BWA-METH, MethylDackel, and Methylation Plotter tools. The study population consisted of MBC (41 cases) compared with gynecomastia (17 cases).

**Results:** *MAGEA* family members, especially *MAGEA2, MAGEA11, MAGEC*, and *UXT* and *HDAC6* showed hypomethylation of several CpGs, reaching statistical significance by the Kruskal–Wallis test (*p* < 0.01) in MBC when compared to gynecomastia. *AR* showed almost no methylation at all.

**Conclusions:** Our study demonstrated for the first time that *MAGEA* family members mapped on the X-chromosome and coregulators of AR are hypomethylated in MBC. This may lead to their overexpression, enhancing AR activity.

## Introduction

Even if its incidence is slightly increasing, male breast cancer (MBC) is a rare disease accounting for about 1% of all breast cancers (1). Due to its rarity, management and treatment are primarily based on postmenopausal female breast cancer (FBC) knowledge. Most MBCs are estrogen receptor (ER) and progesterone receptor (PR) positive and HER2 negative, thus presenting a luminal like profile (2–4) and being considered an ER-driven cancer.

Recent papers based on molecular analyses of large multi-institutional series demonstrated that, in spite of some similarities with FBC, MBC shows a specific molecular portrait (3, 5–7).

Among the genes differentially expressed between MBC and FBC, the androgen receptor (*AR*) gene is emerging as playing a key role in male breast neoplastic transformation (2–4). The importance of *AR* has been demonstrated both on the molecular (8) as well as the morphological basis (4). AR protein, detected by immunohistochemistry, is frequently expressed on MBC, being positive in the large majority of the neoplastic cells (4).

AR maps to the X-chromosome (9). Previous studies performed at our institutions (10, 11) demonstrated X-chromosome polysomy paralleled by AR gene copy number gain in most invasive MBC, as well as in *in situ* carcinoma and in cancer-associated gynecomastia.

On the other side, the gene copy number increase does not necessarily result in higher protein expression. Indeed, CpG islands methylation in gene promoter regions results in gene transcriptional silencing.

In MBC, preliminary data (10) indicated that all additional AR gene copies were hypomethylated, suggesting AR protein overexpression.

*AR* gene expression is modulated by regulators, mainly belonging to melanoma antigen-A11 *(MAGEA11*) family genes, all mapping to the X-chromosome (12).

Therefore, X-chromosome polysomy, which is frequently seen in MBC, can result in a higher gene copy number of *MAGEA11* family genes, therefore causing imbalanced *AR* gene expression modulation. Presently, no data have been published on *MAGEA11* family genes methylation profile in MBC.

Furthermore, gene methylation constitutes an attractive research focus in oncology, often useful to detect prognostic and therapeutically important cancer profiles (13). Due to its rarity, only a few studies focused on MBC methylation profiles (3).

The aim of this study was therefore to evaluate the methylation level of *AR, MAGEA11*, and its family members (*MAGEA2, MAGEC1*, and *MAGEC2*) in MBC. In addition, *AR* regulator genes on the X-chromosome like *FLNA, HDAC6*, and *UXT* were studied. Results obtained in invasive MBC were compared with gynecomastia as controls.

## Materials and Methods

### Patient Collection

MBC and gynecomastia cases were retrieved from the files of the Pathology Units of the Universities of Bologna (at Bellaria Hospital), Rome (at Catholic University, Fondazione Policlinico Universitario A. Gemelli, IRCCS), Italy, Zurich (University Hospital, Institute of Pathology and Molecular Pathology), Switzerland, and Utrecht, The Netherlands. Tissues had been routinely formalin-fixed and paraffin-embedded (FFPE). Cases were retained when enough informative DNA was obtained from the FFPE tissue samples. Gynecomastia cases (*N* = 17) were selected when not associated with invasive carcinoma, either synchronous or metachronous.

All cases were diagnosed according to currently available criteria and had undergone ER, PR, and HER2 immunohistochemical evaluation at the time of diagnosis.

Immunohistochemistry for AR was performed on an automated platform (Ventana, Roche) applying a monoclonal antibody (clone F39.4.1, mouse, BioGenex, San Ramon, CA, USA).

### Ethical Statement

All clinical investigations have been conducted according to the principles expressed in the Declaration of Helsinki. The study was approved by local Ethics Committee of Bologna (protocol number CE-AVEC 17180). Further use of cases was approved by the local ethical committees of Zurich (KEK_2012-553 and KEK-2012-554) and Utrecht (5). All information regarding the human material used in this study was managed using anonymous numerical codes.

### DNA Purification

DNA purification was performed as previously described (14) and summarized as follows. Selected areas containing at least 70% cancer cells were macrodissected by a scalpel starting from 10-μm FFPE sections. The tissue was digested at 56°C for 3 h or overnight using the Quick Extract^TM^ FFPE DNA extraction kit (Epicenter, Madison, WI, USA). After a denaturation step at 95°C for 5 min, the solution was centrifuged at 10,000 × g at 4°C for 5 min. The interphase containing DNA was quantified by Nanodrop (ThermoFisher, MA, USA) and stored at 4°C or immediately processed for the bisulfite-NGS protocol.

### Bisulfite Next-Generation Sequencing

Bisulfite treatment of genomic DNA (100–500 ng) was carried out with the EZDNA Methylation-Lightning™ Kit (Zymo Research, Irvine, CA cod. D5031) according to the manufacturer's protocol. Quantitative DNA methylation analysis was performed as previously described (15) using a two-step PCR protocol for targeted sequencing using the Nextera™ index kit as previously described (16). In brief, well-defined CpG islands of the following 14 genes (see Table 1) were amplified by multiplex PCR: *AR, MAGEA2, MAGEA11, MAGEC1, MAGEC2, FLNA, HDAC6, UXT*, all mapped on the X-chromosome. Locus-specific bisulfite amplicon libraries were generated with tagged primers using Phusion U DNA polymerase (ThermoFisher, cod. F555L) and loaded onto MiSEQ (Illumina, cod. 15027617) according to the manufacturer's protocol. FASTQ output files were processed for quality control (Phred value > 30) and converted into FASTA format in a Galaxy Project environment (17). The DNA methylation level of each CpG was evaluated in parallel using the bisulfite sequencing pattern analysis tool (BSPAT—http://cbc.case.edu/BSPAT/index.jsp) (18), Kismeth (19), and finally BWAMETH followed by the MethylDackel tool in a Galaxy Project environment (Europe) (17). The DNA methylation level of each CpG was compared between MBC and gynecomastia cases by the Kruskal–Wallis *U*-test using the Methylation Plotter tool available online (20). Principal component analysis (PCA) and the methylation HeatMap were created using ClustVis, a web tool for visualizing clustering multivariate data (21).

**Table 1:** Primers coordinates of the seven genes evaluated in this study.

**Gene**	**Description**	**Primer forward**	**Primer reverse**	**Map**	**ENSEMBL**	**Position**	**UCSC h38 coordinates**	**Amplicon length**	**Position respect to TSS**	**Number of interrogated CpGs**
*UXT*	Ubiquitously expressed prefoldin like chaperone	GTTTGGGTGTTTTTGGGTGGT	TCCAATTTAACCTCACACACAATTCAT	Xp11.3	ENSG00000126756	Exon 1	ChrX + strand: 47658973-47659103	130	+78	6
*HDAC6*	Histone deacetylase 6	TTGAGAAAGGGGTTGYGTTT	CTACCCCRTTCCTTCAACCA	Xp11.23	ENSG00000094631	5′UTR/exon1	ChrX + strand: 48801912–48802085	174	−781	15
*AR*	Androgen Receptor	GAGGAGTTTTTTAGAATTTGTTTTAGAG	AAAAACCATCCTCACCCTACTACTAC	Xq11-12	ENSG00000169083	Exon 1	ChrX strand+: 67545205-67545435	231	1,169	9
*MAGEC1*	*MAGE* family member C1	TAGTAGGGTTTAGGGAGTGAGTAGAAA	TCAAAATTAATCAAAACTAACAACCC	Xq27.2	ENSG00000155495	Promoter	ChrX + strand: 141903673-141903833	161	−1,400	7
*MAGEC2*	*MAGE* family member C2	TGTTGGATTTTATTATTTATATTTTTGTTG	AAACTTCCTCCTCTTCCTCATCTATA	Xq27.2	ENSG00000046774	Exon 3	ChrX – strand: 142203870-142204050	181	−63	8
*MAGEA11*	*MAGE* family member A11	GGGAGGATTGAGGTATTTTTATGAT	ACTTCCCTAAATTTACAACAAAAAC	Xq28	ENSG00000185247	intron1-2	ChrX + strand: 149711859-149712030	172	22,883	15
*MAGEA2*	*MAGE* family member A2	TTTTTGTYGTGAATTTAGGGAAG	AATAAAACCCRCCTCAATCC	Xq28	ENSG00000268606	Exon 1	ChrX – strand: 152753752-152753935; ChromX + strand: 152714535-152714718	184	−57	16
*FLNA*	filamin A	TGGAAGAAGATTTAGTAGAATATTTTTA	CTTCTAACTAAACACCTCCAACAAC	Xq28	ENST00000369850.10	Exon2	ChrX – strand: 154370985-154371125	141	+365	12

*Map: chromosome mapping of the gene*.

*Ensembl: Transcript ID in www.ensembl.org~website*.

*UCSC h38 coordinates: chromosome number and human genome 38 coordinates*.

*Position respect to TSS: number of bases from the Transcriptional Start Site*.

## Results

The study population comprised 41 men with a mean age of 63 years (range 49–93). All cases were diagnosed as invasive carcinoma, no special type (IC-NST), and were ER and PR positive. No HER2 amplified cases were included. Clinic-pathological details are reported in Table 2.

**Table 2:** Clinic-pathological details of cases enrolled in this study.

**Male breast cancer**		**Number of cases**
Age	63 (range 49–93)	
Size	T1	35
	T2	4
	T3	1
	T4	1
Lymph-node status	N0	37
	N1	2
	N3	2
Histotype	Invasive carcinoma NST	41
Grade	G1	3
	G2	31
	G3	7
	Number of positive cases	Percentage of positive cells
ER	41	Range 1–100%
PR	36	Range 10–98%
AR	41	Range 10–100%
HER2	0	

**Figure 1:**
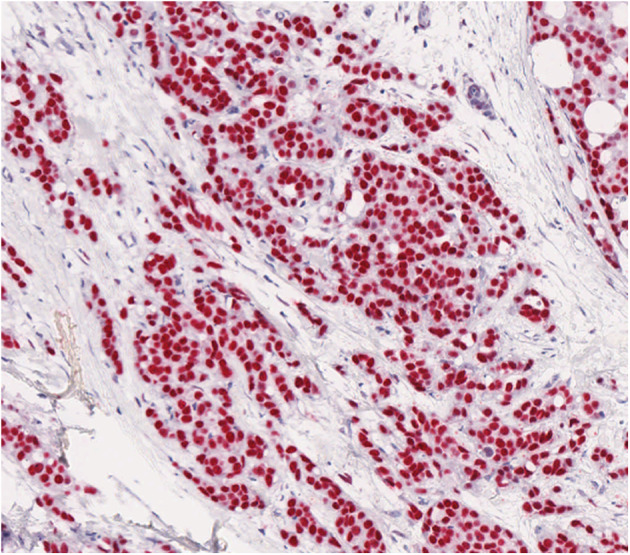
AR shows a strong positivity by immunohistochemistry in most neoplastic cells in male breast cancer.

AR was expressed in all cases. AR antibody stained 10–100% of the neoplastic cells (average 60%, in 86% of the cases, AR was expressed in >60% of the neoplastic cells; Figure 1).

### DNA Methylation Analysis

Bisulfite NGS was used to examine the set of seven genes listed in Table 1, with a total of 92 CpGs, mostly located within the promoter and the first exon.

**Figure 2:**
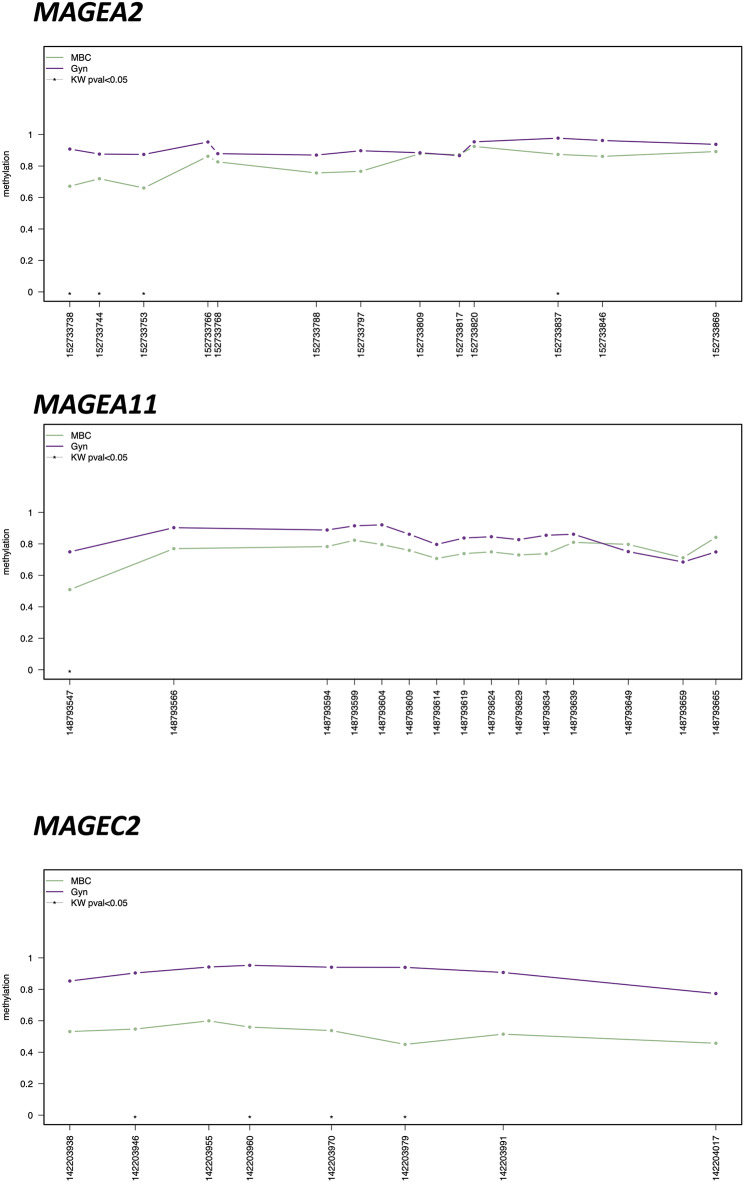
Methylation plotter of *MAGEA2, MAGEA11*, and *MAGEC2*, showing statistically significant differences by Kruskal–Wallis U Test (CpG with KW < 0.05 are highlighted by an asterisk) between male breast cancer (MBC) and gynecomastia (Gyn).

*MAGEA* family members, in particular *MAGEA2, MAGEA11*, and *MAGEC2*, showed the hypomethylation of several CpGs (*p* < 0.01) in MBC compared to gynecomastia (Figure 2). Mean values between MBC and gynecomastia of the most statistically significant CpG of each gene of interest are highlighted in Table 3. *UXT, AR*, and *FLNA* showed at least one statistically significant CpG, but both groups showed methylation levels close to 0 (see Supplementary Table 1 for details).

**Table 3:** DNA methylation mean values between MBC and gynecomastia of the most statistically significant CpG of each gene of interest (^*^p < 0.05).

***Gene***	**Position**	**Gyn methylation mean**	**MBC methylation mean**	**Gyn methylation standard deviation**	**MBC methylation standard deviation**	**Gyn methylation minimum**	**MBC methylation minimum**	**Gyn methylation maximum**	**MBC methylation maximum**	**Kruskall-Wallis *p*-value**
*UXT*	47659074	0.01678235	0.0047439	0.01555885	0.00653242	0	0	0.0411	0.0234	*0.011399
*HDAC6*	48802039	0.01268235	0.00179024	0.02963487	0.00696053	0	0	0.1176	0.0337	0.07347
*AR*	67545280	0	0.0091439	0	0.02121541	0	0	0	0.095	*0.03887
*MAGEC1*	141903813	0.39485	0.2411579	0.2204918	0.2036862	0	0	0.8387	0.7615	0.05113
*MAGEC2*	142203979	0.93955	0.4498455	0.07496397	0.44105528	0.7778	0	1	1	*0.00839
*MAGEA11*	148793547	0.7496625	0.5093436	0.180209	0.2780319	0.4372	0	1	1	*0.001103
*MAGEA2*	152733738	0.9080929	0.6717211	0.04281433	0.24045582	0.8504	0.0081	0.9768	0.9971	*0.0009163
*FLNA*	153599378	0.066	0.00509091	0.09142757	0.00785529	0.004	0	0.171	0.029	*0.034042

*Gyn methylation mean: mean values of methylation values obtained in gynecomastia cases*.

*MBC methylation mean: mean values of methylation values obtained in MBC cases*.

*Gyn methylation standard deviation: range of methylation values obtained in gynecomastia cases*.

*MBC: methylation standard deviation—range of methylation values obtained in MBC cases*.

*Gyn methylation maximum: higher level of methylation obtained in gynecomastia cases*.

*MBC methylation maximum: higher level of methylation obtained in MBC cases*.

**Figure 3:**
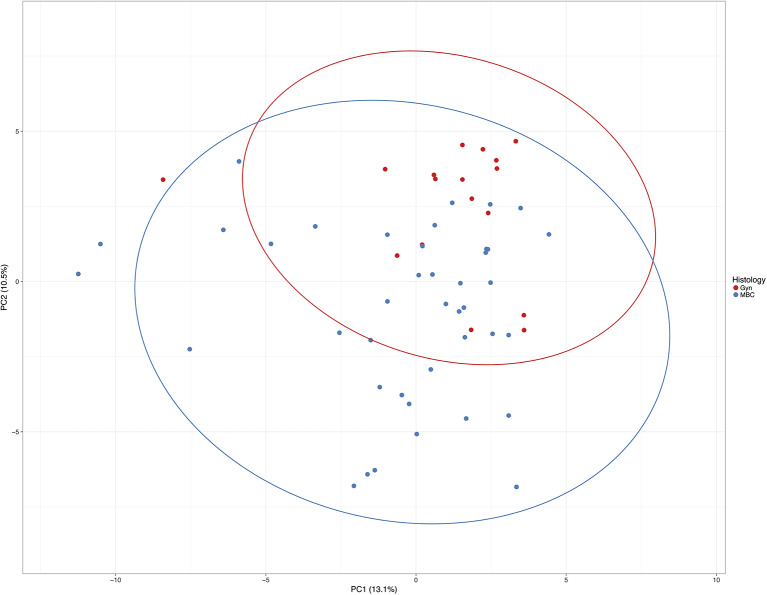
Principle component analysis of all cases included in this study. Unit variance scaling is applied to rows; singular value decomposition (SVD) with imputation is used to calculate principal components. X and Y axes show principal component 1 and principal component 2 that explain 13.1 and 10.5% of the total variance, respectively. Prediction ellipses are such that with probability 0.95, a new observation from the same group will fall inside the ellipse. *N* = 58 data points.

Using the PCA with the highest distribution of data (PC1, x-axis) and the second highest principal component (PC2, the y-axis), cases are distributed considering the methylation level of the total of 92 CpGs (Figure 3).

**Figure 4:**
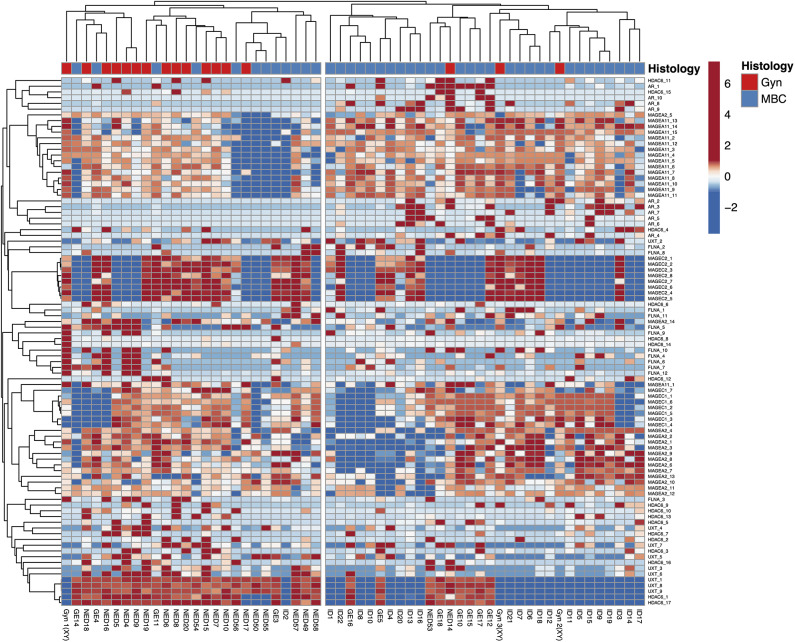
HeatMap using the whole CpGs coming from seven genes evaluated in this study. Rows are centered; unit variance scaling is applied to rows. Both rows and columns are clustered using correlation distance and average linkage; 92 rows, 58 columns.

The HeatMap (Figure 4) generated by data from the whole CpGs coming from seven genes evaluated in this study showed two clusters using correlation distance and average linkage: on the right, 29 MBCs are positioned together with 3 gynecomastia cases; on the left, the remaining 12 MBCs clustered together with 14 gynecomastia cases.

## Discussion

X-chromosome polysomy can be observed in 31 to 85% of the MBC neoplastic cell population (11), being more frequent in IC-NST of higher histological grade and larger size, affecting older men (11). X-chromosome polysomy can be observed in *in situ* carcinoma as well as in cancer-associated gynecomastia (10), thereby suggesting to favor neoplastic transformation.

Several genes that can play a key role in neoplastic transformation of male breast epithelium map to the X-chromosome, like *AR* and its regulators. Therefore, it is plausible that all those genes show copy number gain as a consequence of X-chromosome polysomy. Indeed, results obtained by fluorescent *in situ* hybridization confirm that the *AR* gene copy number parallels the X-chromosome copy number (10, 11).

A higher gene copy number can result in higher protein levels, depending on the CpG island methylation status. Therefore, the methylation profile is crucial to evaluating the functional status of additional gene copies.

Previously published results (10) indicated that *AR* is generally hypomethylated. The results here obtained on a larger number of cases confirm that *AR* is almost completely unmethylated. Therefore, all *AR* gene copies seem to be transcriptionally active.

AR gene expression depends also on several other genes that regulate its function. The AR FXXLF motif region serves as an interaction site for melanoma MAGEA11 (12), a specific *AR* coregulator (22). MAGEA11 increases *AR* transcriptional activity during prostate cancer progression (23). Minges et al. (12) proposed a model in which AR and MAGEA11 form a multidimeric complex where each monomer of the MAGEA11 dimer interacts with an AR FXXLF motif region, forming a bridge between transcriptionally active AR dimers. The *AR* regulators belonging to the *MAGEA11* family also map on the X-chromosome; therefore, they are most likely increased in copy number in MBC.

The results here obtained indicate that all *MAGEA11* family genes are hypomethylated and therefore probably all are transcriptionally active. Several statistically significant CpGs located at the promoter level of *MAGEA2, MAGEA11*, and *MAGEC2* support this hypothesis (see Figure 2 for details). Further studies starting from fresh/frozen tissues are needed to demonstrate overexpression at the RNA and protein level by RNA-SEQ and Western blot analysis, respectively. In fact, these two approaches were not feasible starting from retrospective FFPE tissues, since the nucleic acids are usually very degraded and fragmented. Despite this, we were able to obtain enough DNA from our collection of MBC and gynecomastia, suitable to be processed with sodium bisulfite.

Filamin A (FLNA) regulates the cytoskeleton organization by linking with actin filaments. In addition, FLNA protein interacting with >60 different other proteins can regulate several cell functions ranging from cell migration, transmembrane receptor signaling to DNA damage repair (24, 25). Among the many functions, FLNA interacts with AR reducing its activation in prostate cancer (24). Mooso et al. (25) demonstrated that FLNA inhibits *AR* gene transcription levels; in turn, AR modulates FLNA protein expression and cleavage. As a consequence, FLNA and AR interaction is a key feature in regulating androgen deprivation therapy response in prostate cancer (25).

No data have been previously published on *FLNA* in MBC. According to the present results, the *FLNA* gene extracopies are all hypomethylated and therefore likely transcriptionally active.

Ubiquitously expressed, prefoldin-like chaperone (UXT) mapped to Xp11.23, also called androgen receptor trapped clone-27 (26), is a protein expressed in many different tissues. Among the many functions of the UXT, AR expression reduction in prostate cancer is comprised (24). Only a few data are available on the UXT function in FBC, indicating that UXT modulates *ER* transcriptional activity (27). No further data were available on the interaction between UXT and AR in MBC or FBC. The data shown here, demonstrating *UXT* hypomethylation levels in MBC, suggest that all *UXT* copies are transcriptionally active.

The histone deacetylase 6 (*HDAC6*) mapped to Xp11.23 is an important *AR* regulator in prostatic cancer by enhancing AR protein stability (28). A few data are available in breast cancer. In FBC, a correlation between AR and HDAC6 (29) expression has been demonstrated, especially in those triple negative FBCs showing AR expression. No data were previously published on MBC. The data shown here demonstrate that *HDAC6* is hypomethylated, suggesting an active role in AR signaling.

In summary, the data shown here indicate that AR and its regulators, mapped to the X-chromosome, are hypomethylated in MBC, thus probably being transcriptionally active. All these data are consistent with the fact that AR protein is expressed in the majority of neoplastic cells in MBC, as detected by immunohistochemical methods.

It was not possible to find a relation between immunohistochemical AR expression and AR methylation levels, as most of the cases showed a strong AR positivity in >60% of the neoplastic cells.

Results shown here can be potentially interesting as AR is a therapeutically useful target molecule. Specifically, the present data demonstrate that AR and all its regulators are hypomethylated, but methylation levels vary from case to case. AR and its regulators' methylation levels are not reflected by AR expression evaluated by immunohistochemistry; indeed, to have AR immunohistochemical positivity, a single functioning gene is enough. As discussed above, to obtain a quantitative AR expression evaluation, a Western blot analysis would be necessary. Unfortunately, Western blot analysis is not feasible on a paraffin embedded material but requires freshly frozen tissue. Exact quantitative AR expression evaluation could have an impact on AR deprivation therapy response.

AR deprivation therapy, well-known in prostatic cancer, has been proposed for FBC and MBC with varying results (30). At the moment, only a few studies have been reported (31, 32) focusing on AR deprivation therapy in MBC. Most knowledge is based on single case reports (33). In almost all the reported cases, therapy was proposed on the basis of immunohistochemical AR detection in the neoplastic cells.

In prostate cancer, it has been shown that AR polysomy is associated with castration-therapy resistance (34–37). Similarly, it is plausible that in MBC also, AR deprivation therapy response is related to AR and its coregulators' methylation and functional activity. Therefore, AR and its coregulators' activity should be better investigated to understand the real therapeutic value of anti-AR therapy in MBC.

AR is frequently expressed in FBC. Among triple negative breast cancers, those expressing AR are identified as of the “luminal androgen receptor type” (38), and AR as a possible therapeutic target is under investigation. X-chromosome inactivation (XCI, the so-called lyonization) is well-known in cells of the female body. Female cells have a normal XX chromosomal asset, but one X-chromosome is usually condensed to form the Barr chromatin body. Recently, non-random X-chromosome inactivation and cytosine, adenine, guanine (CAG) repeats on AR genes have been related to increased risk to developing breast cancer (39). Nevertheless, at the best of our knowledge, no data have been published on AR and its regulators' methylation profiles in FBC.

In conclusion, the present study demonstrated for the first time that *MAGEA* family members mapped to the X-chromosome and coregulators of AR are hypomethylated in MBC, reflecting their probable overexpression, which may lead to the enhancement of AR activity. AR and its coregulators' activity may therefore play an important role in AR deprivation therapy response in MBC.

## Data Availability Statement

The original contributions presented in the study are publicly available. This data can be found here: European Nucleotide Archive (accession: PRJEB36374) (https://www.ebi.ac.uk/ena).

## Ethics Statement

The studies involving human participants were reviewed and approved by The study was approved by local Ethics Committee of Bologna (protocol number CE-AVEC 17180). Further use of cases were approved by the local ethical committees of Zurich (KEK_2012-553 and KEK-2012-554). Written informed consent for participation was not required for this study in accordance with the national legislation and the institutional requirements.

## Author Contributions

LMor, RM, and MF contributed to the conception and the design of the study. GG organized the database. LMor and MF wrote the first draft of the manuscript. GG, CM, and LMor performed molecular analyses of the tissues. AMS, MC, and AM contributed to case recruitment and data discussion. AS, GZ, ZV, LMos, and PD revised the cases and contributed to the case selection. All authors contributed to manuscript revision, and read and approved the submitted version.

## Conflict of Interest

MF received funds from Roche and Devicor Mammotome, for course organization and from Biocartis and MSD for professional consultation. None of the funds recevied are related to the present study or influenced the results. The remaining authors declare that the research was conducted in the absence of any commercial or financial relationships that could be construed as a potential conflict of interest.

## Supplementary Material

Supplementary Table 1 DNA methylation data for all the genes of interest, including mean values, standard deviations, minimum and maximum values, Kruskal-Wallis *p* values.
